# Histological validation of fast macromolecular proton fraction mapping as a quantitative myelin imaging method in the cuprizone demyelination model

**DOI:** 10.1038/srep46686

**Published:** 2017-04-24

**Authors:** Marina Yu Khodanovich, Irina V. Sorokina, Valentina Yu Glazacheva, Andrey E. Akulov, Nikolay M. Nemirovich-Danchenko, Alexander V. Romashchenko, Tatyana G. Tolstikova, Lilia R. Mustafina, Vasily L. Yarnykh

**Affiliations:** 1 Research Institute of Biology and Biophysics, Tomsk State University, Tomsk, Russian Federation; 2 Institute of Organic Chemistry, Siberian Branch of the Russian Academy of Sciences, Novosibirsk, Russian Federation; 3 Institute of Cytology and Genetics, Siberian Branch of the Russian Academy of Sciences, Novosibirsk, Russian Federation; 4 Siberian State Medical University, Tomsk, Russian Federation; 5 Department of Radiology, University of Washington, Seattle, WA, United States

## Abstract

Cuprizone-induced demyelination in mice is a frequently used model in preclinical multiple sclerosis research. A recent quantitative clinically-targeted MRI method, fast macromolecular proton fraction (MPF) mapping demonstrated a promise as a myelin biomarker in human and animal studies with a particular advantage of sensitivity to both white matter (WM) and gray matter (GM) demyelination. This study aimed to histologically validate the capability of MPF mapping to quantify myelin loss in brain tissues using the cuprizone demyelination model. Whole-brain MPF maps were obtained *in vivo* on an 11.7T animal MRI scanner from 7 cuprizone-treated and 7 control С57BL/6 mice using the fast single-point synthetic-reference method. Brain sections were histologically stained with Luxol Fast Blue (LFB) for myelin quantification. Significant (p < 0.05) demyelination in cuprizone-treated animals was found according to both LFB staining and MPF in all anatomical structures (corpus callosum, anterior commissure, internal capsule, thalamus, caudoputamen, and cortex). MPF strongly correlated with quantitative histology in all animals (r = 0.95, p < 0.001) as well as in treatment and control groups taken separately (r = 0.96, p = 0.002 and r = 0.93, p = 0.007, respectively). Close agreement between histological myelin staining and MPF suggests that fast MPF mapping enables robust and accurate quantitative assessment of demyelination in both WM and GM.

Macromolecular proton fraction (MPF) is a biophysical parameter defined within the two-pool model of magnetization transfer (MT)^1^ that describes the amount of macromolecular protons characterized by solid-like nuclear magnetic resonance (NMR) spin dynamics and involved into magnetization exchange with free water protons in biological systems. During past decade, MPF has attracted remarkable attention as a quantitative biomarker of myelin due to high sensitivity to demyelination and strong correlations between MPF and myelin content in neural tissues reported in a number of studies^2^,^3^,^4^,^5^,^6^,^7^,^8^,^9^,^10^,^11^,^12^. Associations between MPF and myelination were established in a variety of models and objects including excised demyelinated peripheral nerve^2^, postmortem multiple sclerosis (MS) brain^3^, rat experimental autoimmune encephalomyelitis^4^, shiverer mouse^5^,^6^, lipopolysacharide-induced focal lesion in the rat brain^7^, shaking pup^8^, cuprizone-induced demyelination in mice^9^,^10^, as well as normal rat brain^11^ and spinal cord^12^.

MPF or its associated quantity, pool size ratio (PSR) can be measured by a variety of quantitative MT (qMT) techniques based on semi-selective perturbation of either macromolecular or water proton populations^13^. Common approaches allowing reconstruction of MPF maps from magnetic resonance imaging (MRI) data include Z-spectroscopic imaging based on the analysis of signal dependence on the offset frequency and power of an off-resonance saturation pulse^14^,^15^ and on-resonance techniques based on the analysis of bi-exponential longitudinal relaxation^16^,^17^. These methods allow measurements of several parameters of the two-pool model including MPF and require collection of multiple image sets that results in time-consuming data acquisition rendering them inapplicable in clinics. An alternative fast method allowing whole-brain MPF mapping based on a single MT-weighted image, reference image, and a *T*_1_ map was recently proposed^18^. This technique was tested in multiple sclerosis (MS)^19^ and mild traumatic brain injury^20^ studies and demonstrated a promise as a robust clinically-targeted myelin imaging approach. In the above clinical studies, the fast single-point MPF mapping method^18^ demonstrated the capability to detect subtle microscopic demyelination in normal-appearing brain tissues including both white matter (WM) and gray matter (GM) and revealed the primary clinical relevance of cortical GM demyelination in MS^19^. Time efficiency of single-point MPF mapping has been further improved based on the recent synthetic-reference image reconstruction algorithm^21^,^22^, which requires only three source images and enables generation of three-dimensional (3D) MPF maps with high spatial resolution, whole-brain coverage, and clinically acceptable scan time. While the fast single-point MPF mapping method based on either synthetic^21^,^22^ or actually acquired^18^ reference image showed highly consistent MPF measurements with the standard Z-spectroscopic method in both human^18^ and animal^22^ brain *in vivo*, this approach is still lacking direct histological validation as a quantitative myelin imaging modality.

Cuprizone-induced demyelination in mice is a widely used model of MS that reproduces certain pathological aspects of the disease, such as depletion of oligodendrocytes, myelin degradation, microglial activation, and astrogliosis accompanied with inflammation, hydrocephalus, and brain atrophy^23^,^24^,^25^,^26^. Cuprizone administration typically results in global demyelination of WM and GM with a variable extent across anatomical structures and without formation of macroscopic focal lesions^23^,^24^,^25^,^26^. This feature makes the cuprizone model a very suitable tool for evaluation and validation of quantitative imaging methods intended to measure microscopic demyelination in normal-appearing brain tissues. The overall goal of this study was to validate the fast synthetic-reference MPF mapping method using quantitative histology in the murine cuprizone demyelination model.

## Results

### Comparison between groups

**Figure 1:**
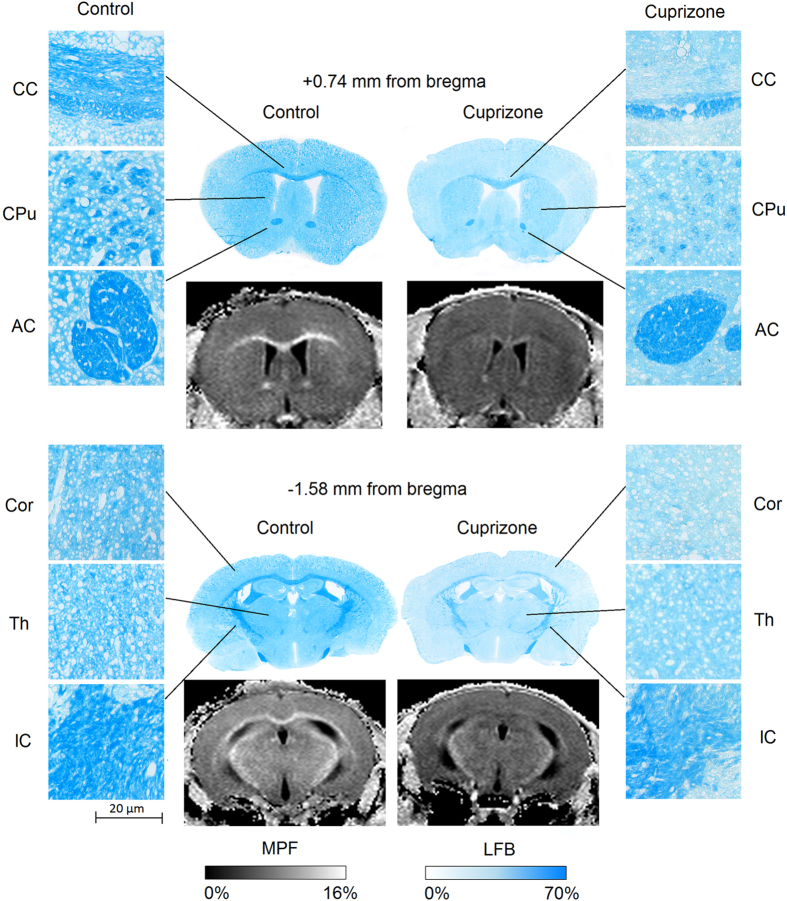
Example coronal brain LFB stained histological sections and cross-sections of 3D MPF maps of the control (left) and cuprizone-treated (right) mice taken through the caudoputamen (+0.74 mm from bregma, top) and thalamus (−1.58 mm from bregma, bottom). Magnified views illustrate normal tissue appearance (left column) and demyelination (right column) on LFB stained sections in the corpus callosum (CC), anterior commissure (AC), internal capsule (IC), caudoputamen (CPu), thalamus (Th), and cerebral cortex (Cor). MPF maps are presented with the grayscale range corresponding to 0–16%. The scale of LFB optical densities corresponds to the range of 0–70%.

**Figure 2:**
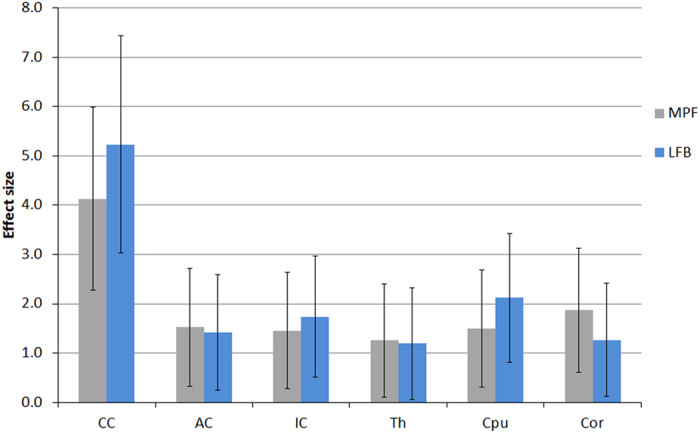
Effect sizes (Cohen’s d) corresponding to group differences in mean MPF and LFB optical densities between control and cuprizone-treated mice for the following brain anatomic structures: corpus callosum (CC), anterior commissure (AC), internal capsule (IC), thalamus (Th), caudoputamen (CPu), and cerebral cortex (Cor). Error bars represent 95% confidence intervals.

Example MPF maps and LFB stained sections of control and cuprizone-treated murine brains are presented in Fig. 1. MPF maps of control animals demonstrated excellent tissue contrast between WM and GM and clear delineation of anatomical structures. Cuprizone-treated mice had visually reduced tissue contrast on MPF maps, especially in the corpus callosum and a slight overall reduction of the image intensity that can be appreciated when compared to control maps presented in the same absolute scale (Fig. 1). A visible reduction in the optical density on LFB stained brain sections of mice treated with cuprizone also can be appreciated in both WM and GM structures (Fig. 1). Quantitative comparison of MPF values and LFB optical densities in a series of anatomical regions between cuprizone-treated and control animals is presented in Table 1. Both MPF and LFB staining intensity in all WM and GM anatomical structures demonstrated a statistically significant decrease in the cuprizone group. Figure 2 compares the effect sizes characterizing group differences across anatomical structures. According to both MPF and LFB data, the largest effect of demyelination was found in the corpus callosum. Despite smaller relative changes in MPF, MPF mapping and LFB staining demonstrated a comparable performance in discriminating cuprizone-treated animals from the control group in terms of the effect size due to smaller variability of MPF measurements.

### Correlations between MPF and histology

**Figure 3:**
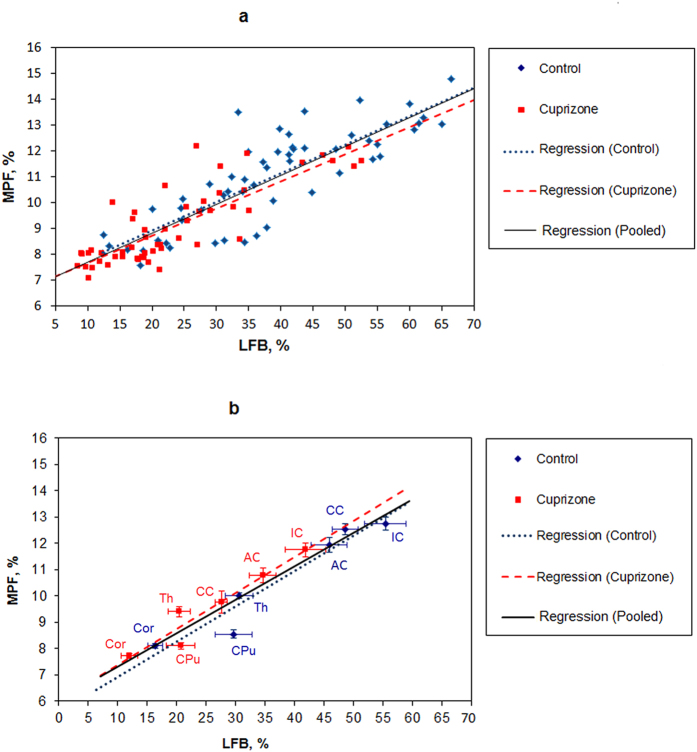
Linear regressions of MPF values on myelin density according to LFB staining in anatomical structures of the murine brain for individual measurements in each animal and structure (**a**) and mean values in each structure (**b**). Blue and red dots correspond to control and cuprizone-treated animals, respectively. Black lines depict the plots of the regression equations for the pooled datasets including both groups. Blue and red lines represent the separate regression plots for the control and cuprizone-treated groups, respectively. Error bars in the panel (b) represent standard errors of mean MPF and LFB optical densities in anatomical structures. Anatomic structures are labeled as follows: corpus callosum (CC), anterior commissure (AC), internal capsule (IC), thalamus (Th), caudoputamen (CPu), and cerebral cortex (Cor).

Results of linear regression analyses of MPF values as a function of LFB optical density for individual and averaged across anatomical structures data are presented in Table 2, and corresponding plots are shown in Fig. 3. In the pooled sample including data for each anatomical structure in each animal at each location from bregma, MPF values and LFB optical densities were strongly correlated (r = 0.88, p < 0.001) (Fig. 3a). This correlation became even stronger (r = 0.95, p < 0.001) after averaging data across animals for each structure (Fig. 3b). Regression analyses for separate groups of cuprizone-treated and control animals demonstrated remarkably similar results in terms of correlation strengths and coefficients of regression equations (Table 2). Particularly, for the subsamples of individual animals and structures, correlation coefficients were r = 0.88 (p < 0.001) for the cuprizone group and r = 0.87 (p < 0.001) for the control group. No significant differences between groups were found for correlation coefficients (p = 0.78) and slopes (p = 0.80) and intercepts (p = 0.79) of the regression lines. Very strong correlations were also observed for average structure data in both groups (r = 0.96, p = 0.002 for cuprizone-treated animals and r = 0.93, p = 0.007 for control animals) with no significant differences in regression coefficients (p = 0.58 for slopes and 0.56 for intercepts) and correlation strengths (p = 0.72).

### Regional distinctions in the corpus callosum demyelination

**Figure 4:**
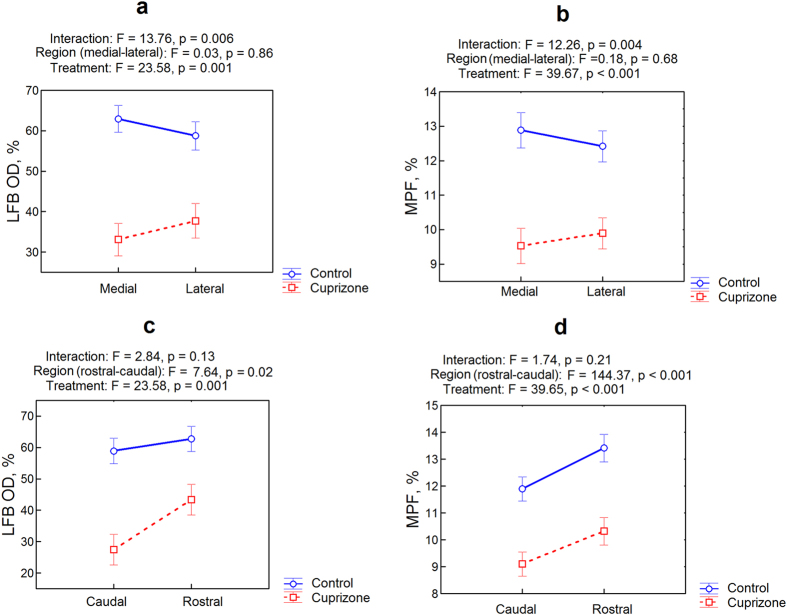
Regional distinctions in the corpus callosum demyelination according to ANOVA based on LFB optical density (**a,c**) and MPF (**b,d**) measurements. Plots (**a**) and (**b**) represent the effect of medial-to-lateral direction. Plots (**c**) and (**d**) represent the effect of caudal-to-rostral direction. Blue and red colors correspond to the control and cuprizone-treated groups, respectively. Error bars represent standard errors.

The extent of demyelination in the corpus callosum was evaluated additionally in the rostral-caudal and medial-lateral directions. The results of the analysis of variance (ANOVA) for different regions of the corpus callosum are presented in Fig. 4. The patterns of the cuprizone treatment effect on regional MPF values and LFB optical density appeared substantially similar (Fig. 4). The significant main effect of treatment was observed for both MPF (F = 39.67, p < 0.001) and LFB optical density (F = 13.76, p = 0.006) measurements in all regions (Fig. 4). In the medial-lateral direction, no significant main effect of the region (F = 0.18, p = 0.68 for MPF and F = 0.03, p = 0.86 for LFB optical density) was observed, but significant interactions between the effects of treatment and region were found for both MPF (F = 12.26, p = 0.004) and LFB (F = 13.76, p = 0.006) data. In the rostral-caudal direction, the significant main effect of the region was identified for both MPF (F = 144.37, p < 0.001) and LFB (F = 7.64, p < 0.02), while interactions between the effects of treatment and region did not reach statistical significance (F = 1.74, p = 0.21 for MPF and F = 2.84, p = 0.13 for LFB). Interactions between factors of the rostral-caudal and medial-lateral direction were also insignificant (F = 0.96, p = 0.35 for MPF and F = 0.03, p = 0.87 for LFB).

### Scan-rescan repeatability of MPF mapping

**Figure 5:**
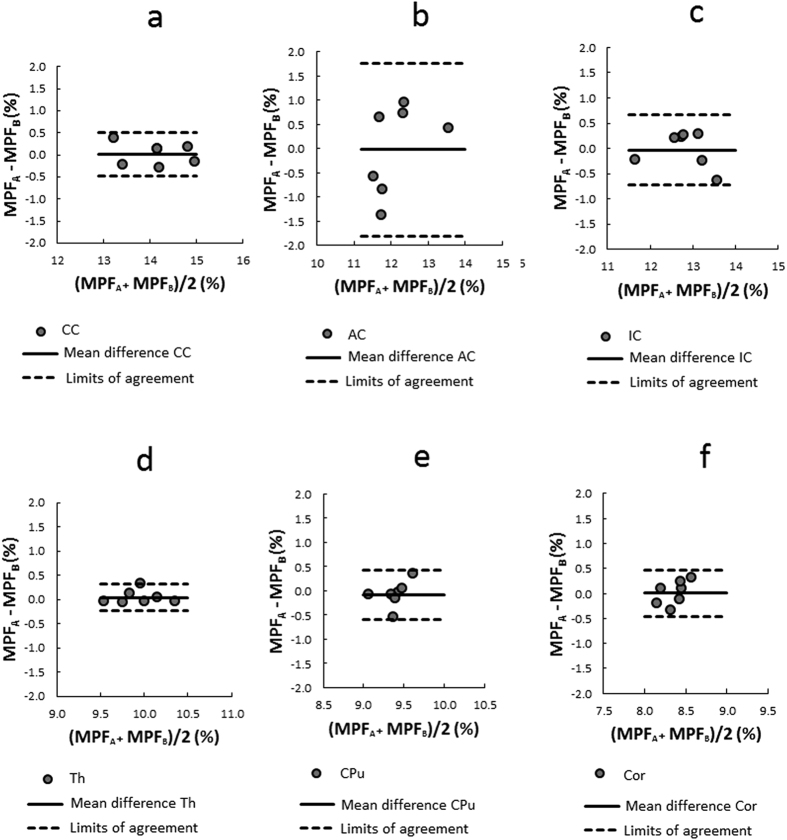
Bland–Altman plots comparing mean MPF values between two consecutive measurements in the following anatomical structures: corpus callosum (**a**), anterior commissure (**b**), internal capsule (**c**), thalamus (**d**), caudoputamen (**e**), and cerebral cortex (**f**). Solid and dashed lines correspond to the mean difference and limits of agreement, respectively.

Bland–Altman plots for repeated MPF measurements in a series of anatomic structures obtained from two consecutive MRI scans are presented in Fig. 5. Detailed report of the statistical analysis of repeatability is provided as a Supplementary Material (Supplementary Table S1). MPF values in all anatomic structures demonstrated close agreement with no significant bias between two measurements (p > 0.1 for all structures) and equal variances within each measurement (p > 0.1 for all structures). Within-subject coefficients of variation (CoV) for specific structures were as follows: 1.2% for the corpus callosum, 4.9% for the anterior commissure, 1.8% for the internal capsule, 1.0% for the thalamus, 1.9% for the caudoputamen, and 1.9% for the cortex.

## Discussion

The key finding of this study is very close agreement between MPF measured by the fast single-point method and quantitative histology in both normal and demyelinated mouse brain across a range of WM and GM anatomical structures. This agreement is confirmed by both strong correlations between MPF and histology and comparable effect sizes for detecting demyelination in both WM and GM. Fast MPF mapping and LFB staining in the corpus callosum also demonstrated very similar capability to measure regional variations in demyelination within a single anatomic structure. The results of this study corroborate earlier reports of strong correlations between MPF and LFB optical density in the normal rodent brain^11^ and the capability of MPF to detect cuprizone-induced demyelination not only in WM but also in cortical GM^9^. Of particular note, the strengths of correlations between MPF and LFB staining across WM and GM structures reported in this study appeared essentially similar to the data for the normal rat brain^11^, as the correlation coefficients larger than 0.9 were found in both studies. It should be emphasized that the patterns and strengths of correlations between MPF and LFB staining discovered in this study were nearly identical for the groups of control and cuprizone-treated animals. This finding suggests that demyelination provides the primary pathological substrate of an MPF decrease in the cuprizone model, whereas potential influences on MPF of other factors that might affect various quantitative MRI measurements (such as inflammation and gliosis) are minor, if not negligible. While not fully generalizable to MPF changes in focal MS lesions in humans due to a possible confounding effect of edema, this conclusion provides a uniform conceptual framework for the interpretation of MPF as a biomarker of demyelination in normal appearing brain tissues. A recent study^19^ have identified a significant decrease of MPF in normal-appearing WM and GM in MS and close associations between MPF in normal-appearing brain tissues and clinical status. Furthermore, MPF changes in normal appearing brain tissues demonstrated a much higher clinical relevance than MPF in lesions or lesion volume^19^. In accordance with a commonly accepted view of MS as a whole-brain disease^27^, the development and validation of sensitive and specific quantitative biomarkers of visually undetectable demyelination in normal-appearing brain tissues may bring both studies of new therapies and individual clinical care in MS to a principally new level. Taken together with earlier pre-clinical and clinical observations^9^,^11^,^19^,^20^, the results of this study suggest that MPF provides a robust quantitative measure of the myelin content in both WM and GM, while the technical simplicity, short scan time, and whole-brain coverage enabled by the single-point synthetic-reference MPF mapping method^21^,^22^ make MPF a sensible biomarker for a variety of pre-clinical and clinical studies.

Cuprizone-induced demyelination of WM in mice has been studied by a variety of MRI methods^9^,^10^,^28^,^29^,^30^,^31^,^32^,^33^,^34^,^35^,^36^,^37^,^38^,^39^,^40^,^41^,^42^. A vast majority of these studies were primarily focused on the corpus callosum, the site of most prominent myelin loss^23^,^24^,^25^,^26^, which can be easily detected by routine *T*_2_- and *T*_1_-weighted imaging^28^,^29^,^30^,^31^ and a number of quantitative techniques including diffusion tensor imaging (DTI)^9^,^29^,^32^,^33^,^34^,^35^,^36^,^37^,^38^,^39^, conventional MT ratio (MTR) imaging^9^,^28^,^34^,^36^,^40^,^41^,^42^, qMT^9^,^10^, and multi-component relaxation analysis^38^. Several MRI studies also investigated demyelination in other WM structures, such as cerebral peduncles^32^, anterior commissure^32^, optic tracts^32^, external capsule^9^,^10^,^30^,^32^, and cingulum^30^,^32^ which are affected by cuprizone intoxication to a lesser extent^32^,^43^. Of note, demyelination of the corpus callosum in the cuprizone model exhibits substantial spatial and temporal variability^29^,^39^,^42^,^43^,^44^,^45^,^46^. Both MPF mapping and histology in our study confirmed the presence of the medial-to-lateral gradient of demyelination that is in agreement with the earlier publication^42^. However, in contrast to previous studies^29^,^39^,^42^,^43^,^44^,^45^,^46^, we did not find significant distinctions in the myelin loss in the rostral-caudal direction. The most probable explanation of this discrepancy is a substantially longer cuprizone treatment period (8 weeks) in our study compared to earlier ones where the rostral-caudal gradient of demyelination was detected after 4–6 weeks of cuprizone administration^29^,^39^,^42^,^43^,^44^,^45^,^46^. As such, regional distinction in the myelin content observable within a certain time frame might have been equalized due to either more extensive demyelination or concurrent remyelination^23^,^39^,^46^. In agreement with this explanation, a reduction of rostral-caudal differences in demyelination was observed during prolonged cuprizone treatment (up to12 weeks)^39^. Another possible reason is inherent spatial variations of the myelin content in the normal corpus callosum as indicated by both MPF mapping and LFB staining (Fig. 4), which might mask relatively subtle regional differences in the demyelination. While spatial patterns of cuprizone-induced demyelination generally have a complex dependence on the treatment dose, duration, and mouse strain^29^,^39^,^42^,^43^,^44^,^45^,^46^, our study indicates that MPF provides nearly identical to histology results in quantifying a variable extent of myelin loss in WM.

Cortical and subcortical GM demyelination is a characteristic feature of the cuprizone model according to histological studies^47^,^48^,^49^,^50^,^51^, though MRI data about GM in cuprizone-treated animals are scarce. Particularly, a decrease of MTR was reported in deep GM^41^, while MTR could not distinguish cortical demyelination between treatment and control groups^9^,^34^,^41^. A quantitative parameter based on multicomponent *T*_2_ relaxation, myelin water fraction^52^,^53^, which has been proposed as a myelin biomarker, was unable to detect both cortical and subcortical GM demyelination, while showing a significant effect in the corpus callosum and cerebellar WM in a recent cuprizone murine *ex vivo* study^38^. Quantitative indexes derived from DTI data, such as fractional anisotropy and radial diffusivity were sensitive to cuprizone-induced changes in WM^9^,^29^,^32^,^33^,^34^,^35^,^36^,^37^,^38^ but failed to identify distinctions in GM^9^,^37^. A more sophisticated diffusion-based technique, diffusion kurtosis imaging (DKI) demonstrated quantitative changes in the cortex of cuprizone-treated mice^37^. However, these changes appeared inconsistent with changes in the corpus callosum, thus suggesting a complex mechanism determining a behavior of DKI parameters that cannot be reduced to demyelination alone^37^. Significant cortical changes in cuprizone-treated mice were reported for MPF, cross-relaxation rate constant, and *T*_1_ based on the analysis of Z-spectroscopic qMT data^9^. To the best of our knowledge, none of previous cuprizone model studies quantitatively compared any MRI technique with histology in the context of absolute myelin content measurements across a range of anatomical structures including WM and both cortical and subcortical GM. This study demonstrates that MPF measurements based on the fast single-point synthetic-reference method^21^ provide a similar to quantitative histology performance in the assessment of both WM and GM demyelination. A number of post-mortem histological studies have indicated a considerable amount of cortical and subcortical GM demyelination in MS in addition to WM damage^27^,^54^,^55^,^56^,^57^. High clinical relevance of cortical demyelination in MS has been established in a recent study^19^, where MPF in cortical GM was found to be the strongest predictor of disability and the secondary progressive MS disease phenotype^19^. As such, the histologically validated capability of fast MPF mapping to accurately quantify GM demyelination makes this method especially attractive from the clinical standpoint.

Another useful feature of fast MPF mapping in the context of both pre-clinical and clinical studies is high reliability of results. Our study of scan-rescan repeatability has demonstrated excellent performance of the method in both WM and GM structures. For MPF measurements in all explored anatomical structures, except for the anterior commissure, within-subject CoV did not exceed 2%. The MPF values in the anterior commissure had a higher variability with CoV of about 5% that is probably due to the small size and associated partial volume effect for this structure. Repeatability of fast MPF mapping in the murine brain approaches earlier data for the human brain^58^ where within-subject CoV of 1.5 and 1.1% were reported for WM and GM, respectively^58^. Slightly larger CoV in the present work can be addressed to the manual procedure of image analysis as opposed to global brain tissue segmentation in the human study^58^. At the same time, both this and previous^58^ studies indicate that the effect of instrumental factors on variability of MPF measurements is minimal.

Compared to other quantitative MRI techniques proposed for myelin characterization, fast MPF mapping offers several practical advantages. An empirical value describing the MT effect, MTR has been widely used to characterize demyelination^59^. While sensitivity of MTR to myelin mainly originates from its dependence on MPF^11^,^15^,^19^, MTR also depends on *T*_1_. MPF and *T*_1_ contribute into MTR in opposite directions, thus reducing its sensitivity to myelin content variations^15^,^19^. Additionally, the dependence on *T*_1_ makes MTR sensitive to the magnetic field strength and the effect of paramagnetic ions, such as iron, which frequently confounds demyelination, particularly in subcortical GM^57^,^60^,^61^. MPF itself is independent of the presence of paramagnetic substances and field strength^22^. Alternative semi-empirical indexes describing the MT effect, such as MT saturation^62^, and MTR difference observed in the recent inhomogeneous MT experimental method^63^ were proposed to overcome limitations of MTR. While the maps of the above quantities provide similar to MPF brain tissue contrast, these techniques have not been histologically validated, and the feasibility of their use for absolute quantitation at different field strengths remains uncertain. In contrast to DTI parameters associated with myelination (fractional anisotropy and radial diffusivity)^32^,^33^,^34^, MPF is insensitive to directional properties and spatial organization of WM fiber tracts, which inevitably confound DTI-based measurements and result in high anatomical heterogeneity of corresponding indices that is unrelated to the myelin content^64^,^65^,^66^. In contrast to relaxation-based methods aimed to extract myelin water fraction from multi-component *T*_2_^52^, *T*_2_*^67^, or combined *T*_1_ and *T*_2_ data analysis^53^, MPF is insensitive to errors caused by iron deposition^21^,^68^ and variability of relaxation times associated with magnetic field strength^22^. MPF also has been shown to provide a more accurate measure of myelin than multi-component *T*_2_, since the latter is subjected to systematic errors caused by inter-compartmental water exchange^12^. A new relaxation-based method, relaxation along a fictitious field (RAFF) has recently emerged and showed a promise for myelin quantitation based on the comparisons with histology^69^. However, it remains unclear at this point, whether quantitative data obtained with this approach depend on magnetic field and paramagnetic contributions and whether a RAFF mapping technique can provide sufficient time efficiency and image quality for clinical applications. Finally, MPF maps obtained by the fast single-point synthetic-reference method^21^,^22^ offer a substantially better spatial resolution and image quality *in vivo* than parametric maps typically available within DTI, multi-component relaxation, and multi-parameter qMT methods.

## Conclusions

Fast 3D MPF mapping provides a robust clinically-targeted quantitative myelin imaging modality that showed the capability to detect demyelination in normal-appearing brain tissues in recent MS and mild traumatic brain injury studies. This study provides the first quantitative histological validation of the fast MPF mapping method in the animal demyelination model. Very strong correlations between histological myelin staining and MPF suggest that the method enables accurate quantitative assessment of the myelin content in both WM and GM and that demyelination is a major or even sole pathological substrate of MPF changes in demyelinating diseases.

## Methods

### Animal procedures and histological processing

All animal experiments were performed in accordance with the rules adopted by the European Convention for the Protection of Vertebrate Animals used for Experimental and other Scientific Purposes. The experimental protocol was approved by the Bioethical Committee of the Institute of Cytology and Genetics of the Siberian Branch of the Russian Academy of Sciences and the Bioethical Committee of the Biological Institute at the Tomsk State University. Fourteen five-week-old C57BL/6 male mice were obtained from the vivarium of the N.N.Vorozhtzov Institute of Organic Chemistry (Novosibirsk, Russian Federation). Animals were housed with a 12 hours dark-light cycle at the temperature of 21 ± 2 °C, and humidity of 40 ± 2%. Food and water were provided ad libitum. Seven mice were treated 0.3% cuprizone solution in drinking water during 8 weeks to induce demyelination, and seven control mice received regular vivarium chow and water. Imaging was performed under isoflurane anesthesia (1.5–2% in oxygen) with respiratory monitoring during the scan.

After scanning, mice were transcardially perfused with 4% paraformaldehyde (PFA) under ether anesthesia. The brains were removed and fixed overnight in PFA at 4 °C. Then the brains were cryoprotected in graded concentration of sucrose in phosphate buffer (24 h at 10% and 24 h at 20%) at 4 °C, frozen in liquid nitrogen, and stored at −80 °C. Coronal brain sections with 10 μm thickness were prepared using an HM525 cryostat (Thermo Fisher Scientific, Walldorf, Germany). Histological myelin staining was performed using Luxol Fast Blue (LFB) colorant (Bio-Optica, Milano, Italy). Whole-brain sections were photographed using an Axio Imager Z2 microscope (Carl Zeiss AG, Göttingen, Germany) with the EC PlanNeofluar lx/0.025 objective and AxioVision 4.8 software. Identical imaging parameters (voltage of microscope lamp and exposure time) were set for all photographed slices.

Additional seven eight-week-old C57BL/6 male mice were used to assess scan-rescan repeatability of MPF mapping. Animals were kept under standard vivarium conditions, as specified above, and scanned twice with one week interval between scans.

### MRI acquisition and processing

Mice were imaged on an 11.7 Tesla horizontal-bore animal MRI scanner (BioSpec 117/16 USR; Bruker BioSpin, Ettlingen, Germany) with manufacturer’s four-channel mouse brain surface coil. Imaging was performed under isoflurane anesthesia (1.5–2% in oxygen) with respiratory monitoring during the scan. A fast high-resolution 3D MPF mapping protocol was implemented according to the previously described single-point method with synthetic reference image normalization^21^,^22^, and scan parameters were adapted for mouse whole-brain imaging. The protocol included the following sequences applied in the coronal plane with 3D field-of-view of 20 × 20 × 24 mm:

1. MT-weighted spoiled gradient echo (GRE) imaging: repetition time (TR) = 22 ms, echo time (TE) = 2.5 ms, flip angle (FA) = 9°, spectral bandwidth (BW) = 125 kHz, off-resonance saturation by the Gaussian pulse with offset frequency of 4.5 kHz, effective FA = 900°, and duration of 10 ms, 3D matrix 200 × 200 × 48, spatial resolution 100 × 100 × 500 μm^3^, four signal acquisitions, scan time 10 min 34 s;
2. *T*_1_-weighted spoiled GRE imaging: TR = 16 ms, TE = 2.5 ms, FA = 16°, BW = 125 kHz, 3D matrix 200 × 200 × 48, spatial resolution 100 × 100 × 500 μm^3^, four signal acquisitions, scan time 7 min 41 s;
3. Proton-density (PD)-weighted spoiled GRE imaging: TR = 16 ms, TE = 2.5 ms, FA = 3°, BW = 125 kHz, 3D matrix 200 × 200 × 48, spatial resolution 100 × 100 × 500 μm^3^, four signal acquisitions, scan time 7 min 41 s;
4. *B*_0_ mapping based on the dual-TE GRE phase-difference method^70^: TR = 20 ms, TE_1_ = 2.4 ms, TE_2_ = 4.1 ms, FA = 8°, BW = 200 kHz, 3D matrix 100 × 100 × 48, spatial resolution 200 × 200 × 500 μm^3^, one signal acquisition, scan time 1 min 36 s.
5. *B*_1_ mapping based on the dual-TR actual flip-angle imaging (AFI) method^71^: TR_1_ = 13 ms, TR_2_ = 65 ms, TE = 3.7 ms, FA = 60°, BW = 59.5 kHz, 3D matrix 100 × 100 × 48, spatial resolution 200 × 200 × 500 μm^3^, one signal acquisition, scan time 4 min 45 s.

The GRE and AFI sequences were implemented with optimal radiofrequency and gradient spoiling based on the excitation pulse phase increments of 169° for GRE and 39° for AFI^72^. In all sequences, linear phase-encoding order with fractional (75%) *k*-space acquisition in the slab selection direction was used. The total protocol execution time was about 35 min.

Reconstruction of MPF maps was carried out using custom-written C-language software based on the algorithm detailed elsewhere^21^. In brief, this algorithm computes MPF maps from three source images (MT-, *T*_1_-, and PD-weighted) with correction of *B*_0_ and *B*_1_ field non-uniformities by voxel-based iterative single-point solution of the matrix pulsed MT equation^18^ with a calculated synthetic reference image for data normalization^21^.

The same protocol and map reconstruction was implemented to examine reproducibility of MPF mapping.

### Image analysis

**Figure 6:**
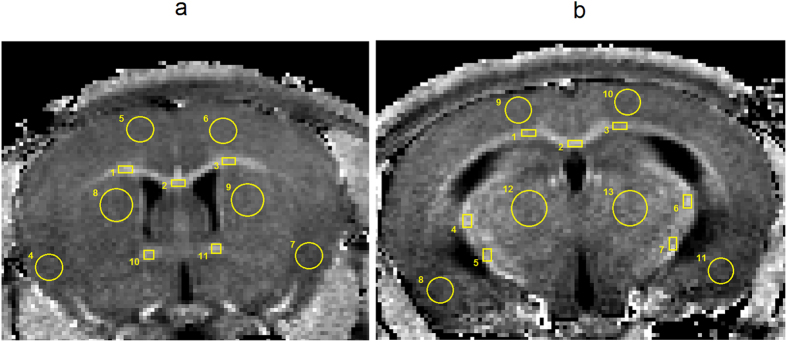
Representative cross-sections of a 3D MPF map of a control mouse taken at two locations from bregma with the superimposed sets of ROIs within investigated WM and GM brain structures. (**a**) Location at +0.74 mm from bregma with ROI numbers corresponding to the following structures: 1, 2, 3 - corpus callosum; 4, 5, 6, 7 - cerebral cortex; 8, 9 – caudoputamen; 10, 11 – anterior commissure. (**b**) Location at −1.58 mm from bregma with ROI numbers corresponding to the following structures: 1, 2, 3 - corpus callosum; 4, 5, 6, 7 - internal capsule; 8, 9, 10, 11 - cerebral cortex; 12, 13 - thalamus.

MPF maps and microphotographs of LFB stained histological sections were analyzed using publically available ImageJ software (National Institutes of Health, Bethesda, MD, USA). Two brain locations (−1.58 mm and +0.74 mm from bregma) defined according to a mouse brain atlas^73^ were chosen for quantitative analysis. MPF values and LFB optical densities were measured for a series of WM and GM structures including corpus callosum, anterior commissure, internal capsule, thalamus, caudoputamen, and cortex. Regions-of-interest (ROIs) of a standard size and shape were manually placed on MPF maps and photographs of LFB stained sections within investigated brain structures using a mouse brain atlas^73^ as reference. The scheme of ROI measurements is exemplified in Fig. 6. MPF values for each anatomical structure were quantified by averaging ROI measurements obtained from 3D MPF maps corresponding to each location from bregma. Myelin densities on LFB stained photographs were quantified using the procedure modified from that described by Underhill, *et al*.^11^. The mean intensity of the red channel (*I*_R_) in a ROI was measured from RGB images as a quantity characterizing the complementary blue channel saturation. Specifically, visual perception of a more saturated blue color reflects stronger light absorption in the red region of the spectrum associated with stained myelin that translates into a lower intensity of the red channel in RGB images. Additionally, the mean intensity of the red channel in the background was measured on each photograph in four ROIs outside the brain. The intensities from the four above ROIs were averaged and used as the background correction factor (*I*_B_). LFB optical density (in %) was subsequently calculated for each ROI as 100 × [1 − (*I*_R_/*I*_B_)], which yields a higher myelin content for increasing values. Individual ROI measurements were obtained from three adjacent sections per each location from bregma and averaged for each anatomical structure.

### Statistical analysis

All statistical analyses were carried out in Statistica 10.0 for Windows (StatSoft Inc, Tulsa, OK, USA). Mean values and standard deviations (SD) of MPF and LFB optical density were calculated for each anatomical structure. Normality of the data within animal groups and residuals in regression analyses was assessed using Shapiro-Wilk test. No significant deviations from the normal distribution were found, and therefore, parametric analyses were used. MPF values and quantitative histology data were compared between cuprizone-treated and control animals using one-way multivariate analysis of variance (MANOVA) followed by independent-samples t-tests for individual anatomical structures. Distinctions in variables between animal groups were described by the effect size (Cohen’s d) calculated as the ratio of the mean difference to the pooled standard deviation.

Pearson’s correlation coefficient (r) and linear regression analysis were used to determine associations between MPF values and LFB optical densities across anatomical structures and animals. Significance of differences between Pearson’s correlation coefficients for the two groups of animals was tested using Fisher’s r-to-z transformation. Slopes and intercepts of regression equations were compared between groups using analysis of covariance (ANCOVA)^74^ where MPF values were used as a dependent variable, LFB optical density was used as a covariate, and group membership (control or cuprizone-treated) was used as a categorical factor.

Regional distinctions in the corpus callosum demyelination were assessed using three-way analysis of variance (ANOVA) with the factors of treatment, ROI location in the rostral-caudal direction, and ROI location in the medial-lateral direction. The model included the main effects of factors and their pairwise interactions.

An agreement between MPF maps obtained from two repeated scans was assessed using Bland–Altman plots for each anatomical structure. Significance of the bias between repeated MPF measurements was examined using the one sample t-test for the mean difference. The limits of agreement were calculated as the mean difference ±1.96 SD of the mean difference. To estimate variability between repeated measurements, the within-subject coefficients of variation was calculated as the percentage ratio of SD to the mean of paired measurements. The Levene’s test was used to assess equality of variances. Two-tailed tests were used in all analyses. Statistical significance was defined as a p value less than 0.05.

## Additional Information

**How to cite this article**: Yu. Khodanovich, M. *et al*. Histological validation of fast macromolecular proton fraction mapping as a quantitative myelin imaging method in the cuprizone demyelination model. *Sci. Rep.* **7**, 46686; doi: 10.1038/srep46686 (2017).

**Publisher's note:** Springer Nature remains neutral with regard to jurisdictional claims in published maps and institutional affiliations.

## Supplementary Material

Supplementary Table S1

## Figures and Tables

**Table 1:** Mean values and standard deviations of MPF and LFB optical densities in the brain anatomical structures of control (n = 7) and cuprizone-treated (n = 7) mice.

Parameter	MANOVA	Structure	Control (mean ± SD)	Cuprizone (mean ± SD)	p
MPF (%)	>Wilks’ Lambda = 0.047, F = 23.35, p < 0.001	Corpus callosum	13.15 ± 0.73	9.65 ± 0.95	<0.001
		Anterior commissure	11.93 ± 0.74	10.78 ± 0.77	0.014
		Internal capsule	12.74 ± 0.67	11.75 ± 0.69	0.018
		Thalamus	10.01 ± 0.44	9.41 ± 0.51	0.035
		Caudate putamen	8.54 ± 0.25	8.10 ± 0.33	0.016
		Cortex	8.13 ± 0.22	7.67 ± 0.27	0.005
>LFB OD (%)	>Wilks’ Lambda = 0.101, F = 10.40, p = 0.003	Corpus callosum	49.14 ± 3.23	29.35 ± 4.27	<0.001
		Anterior commissure	46.09 ± 7.61	37.48 ± 4.01	0.021
		Internal capsule	52.54 ± 8.43	39.92 ± 5.87	0.007
		Thalamus	25.76 ± 3.69	20.59 ± 4.90	0.046
		Caudate putamen	30.68 ± 6.21	18.64 ± 5.09	0.002
		Cortex	16.46 ± 3.40	12.21 ± 3.30	0.035

Abbreviations: MANOVA, multivariate analysis of variance; SD, standard deviation; MPF, macromolecular proton fraction; LFB, luxol fast blue; OD, optical density.

**Table 2:** Linear regression analysis of associations between MPF values and LFB optical densities.

Animal data	Sample	r	r^2^	n	p	Slope (95% CI), p	Intercept (95% CI), p
>Individual	Pooled	0.882	0.779	112	<0.001	0.124 (0.112, 0.137), <0.001	6.278 (5.860, 6.670), <0.001
	Control	0.866	0.750	56	<0.001	0.121 (0.105, 0.145), <0.001	6.243 (5.476, 7.009), <0.001
	Cuprizone	0.879	0.772	56	<0.001	0.125 (0.104, 0.139), <0.001	6.300 (5.751, 6.986), <0.001
Structure-averaged	Pooled	0.947	0.897	12	<0.001	0.135 (0.102, 0.167), <0.001	5.941 (4.854, 7.027), <0.001
	Control	0.932	0.870	6	0.007	0.139 (0.099, 0.178), 0.007	5.650 (2.736, 8.564), 0.006
	Cuprizone	0.962	0.927	6	0.002	0.135 (0.083, 0.188), 0.002	5.996 (4.513, 7.480), <0.001

Abbreviation: CI, confidence interval.
