# Supplementary material for: Histological validation of fast macromolecular proton fraction mapping as a quantitative myelin imaging method in the cuprizone demyelination model
Source: Sci Rep. 2017 Apr 24;7:46686. doi: 10.1038/srep46686 (PMC5402392; doi:10.1038/srep46686)
Supplement: Supplementary Table S1 [file srep46686-s1.doc]

**Histological validation of fast macromolecular proton fraction mapping as a quantitative myelin imaging method in the cuprizone demyelination model**

Marina Yu. Khodanovich^1^ , Irina V. Sorokina^2^, Valentina Yu. Glazacheva^1^, Andrey E. Akulov^1, 3^, Nikolay M. Nemirovich-Danchenko^1^, Alexander V. Romashchenko^3^, Tatyana G. Tolstikova^2^, Lilia R. Mustafina^4^, & Vasily L. Yarnykh^1, 5*^.

^1^Research Institute of Biology and Biophysics, Tomsk State University, Tomsk, Russian Federation;

^2^Institute of Organic Chemistry, Siberian Branch of the Russian Academy of Sciences, Novosibirsk, Russian Federation;

^3^Institute of Cytology and Genetics, Siberian Branch of the Russian Academy of Sciences, Novosibirsk, Russian Federation;

^4^Siberian State Medical University, Tomsk, Russian Federation;

^5^Department of Radiology, University of Washington, Seattle, WA, United States.

^*^Correspondence to: Vasily L. Yarnykh, PhD, Email: [yarnykh@uw.edu](mailto:yarnykh@uw.edu)

**Supplementary Information**

| Value | CC | AC | IC | Th | CPu | Cor |
| --- | --- | --- | --- | --- | --- | --- |
| Mean MPF ± SD,  1^st^ scan (%) | 13.64 ± 0.69 | 12.16 ± 0.63 | 12.83 ± 0.68 | 9.93 ± 0.26 | 9.43 ± 0.14 | 8.37 ± 0.10 |
| Mean MPF ± SD,  2^nd^ scan (%) | 13.65 ± 0.63 | 12.14 ± 1.01 | 12.81 ± 0.60 | 9.97 ± 0.29 | 9.35 ± 0.27 | 8.37 ± 0.25 |
| Mean MPF difference ± SD  (bias) (%) | 0.02 ± 0.25 | -0.02 ± 0.91 | -0.03 ± 0.35 | 0.04 ± 0.14 | -0.08 ± 0.26 | 0.00 ± 0.24 |
| Limits of  agreement (%) | -0.48; 0.51 | -1.80; 1.77 | -0.72; 0.66 | -0.24; 0.32 | -0.59; 0.44 | -0.47; 0.47 |
| Significance for bias (p) | 0.9 | 1.0 | 0.8 | 0.5 | 0.5 | 1.0 |
| Significance for inequality of variances (p) | 0.9 | 0.1 | 0.6 | 0.6 | 0.3 | 0.1 |
| Within-subject CoV (%) | 1.20 | 4.91 | 1.81 | 0.97 | 1.92 | 1.87 |

| **Supplementary Table S1. Analysis of scan-rescan repeatability of MPF measurements.** Abbreviations: CC, corpus callosum; AC, anterior commissure; IC, internal capsule; Th, thalamus; CPu, caudoputamen; Cor, cerebral cortex; MPF, macromolecular proton fraction; SD, standard deviation; CoV, coefficient of variation. |
| --- |
